# 9-Phenyl-3,6-bis­(4,4,5,5-tetra­methyl-1,3,2-dioxaborolan-2-yl)-9*H*-carbazole

**DOI:** 10.1107/S1600536811025645

**Published:** 2011-07-06

**Authors:** Weibing Wu, Jinan Tang

**Affiliations:** a Jiangsu Provincial Key Laboratory of Pulp & Paper Science & Technology, College of Light Industry Science and Engineering, Nanjing Forestry University, Nanjing 210037, People’s Republic of China; b College of Chemistry and Chemical Engineering, Southeast University, Nanjing 211189, People’s Republic of China

## Abstract

In the title compound, C_30_H_35_B_2_NO_4_, the carbazole skeleton is essentially planar (r.m.s. deviation for all non-H atoms = 0.035 Å), and is oriented at a dihedral angle of 65.0 (3)° with respect to the adjacent phenyl ring.

## Related literature

The title compound is an inter­mediate in the synthesis of 9-phenyl­carbazole-based optical materials, see: Oliveira *et al.* (2005 ▶). For the synthesis of the title compound, see: Wong *et al.* (2005 ▶, 2006 ▶); Rashidnadimi *et al.* (2008 ▶). For related structures, see: Xu *et al.* (2010 ▶); Cui *et al.* (2009 ▶); Saeed *et al.* (2010 ▶). For standard bond lengths, see: Allen *et al.* (1987 ▶).
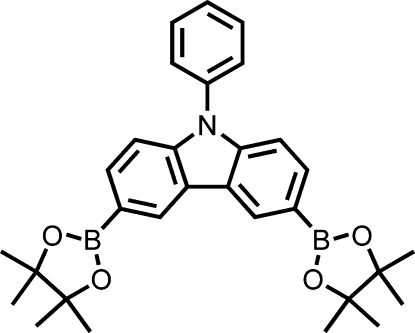


## Experimental

### 

#### Crystal data

C_30_H_35_B_2_NO_4_
*M* *_r_* = 495.21
Orthorhombic, 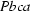

*a* = 13.974 (6) Å
*b* = 11.935 (5) Å
*c* = 34.494 (14) Å
*V* = 5753 (4) Å^3^
*Z* = 8
Mo *K*α radiation
μ = 0.07 mm^−1^
*T* = 298 K
0.3 × 0.2 × 0.1 mm

#### Data collection

Rigaku Mercury2 diffractometer
Absorption correction: multi-scan (*CrystalClear*; Rigaku, 2005 ▶) *T*_min_ = 0.9, *T*_max_ = 1
49645 measured reflections
6553 independent reflections
5170 reflections with *I* > 2σ(*I*)
*R*_int_ = 0.062

#### Refinement

*R*[*F* ^2^ > 2σ(*F* ^2^)] = 0.093
*wR*(*F* ^2^) = 0.242
*S* = 1.16
6553 reflections
342 parameters
15 restraints
H-atom parameters constrained
Δρ_max_ = 0.72 e Å^−3^
Δρ_min_ = −0.52 e Å^−3^

### 

Data collection: *CrystalClear* (Rigaku, 2005 ▶); cell refinement: *CrystalClear*; data reduction: *CrystalClear*; program(s) used to solve structure: *SHELXS97* (Sheldrick, 2008 ▶); program(s) used to refine structure: *SHELXL97* (Sheldrick, 2008 ▶); molecular graphics: *SHELXTL* (Sheldrick, 2008 ▶); software used to prepare material for publication: *SHELXTL*.

## Supplementary Material

Crystal structure: contains datablock(s) I, global. DOI: 10.1107/S1600536811025645/jh2305sup1.cif

Structure factors: contains datablock(s) I. DOI: 10.1107/S1600536811025645/jh2305Isup2.hkl

Supplementary material file. DOI: 10.1107/S1600536811025645/jh2305Isup3.cml

Additional supplementary materials:  crystallographic information; 3D view; checkCIF report

## Figures and Tables

**Fig. 1.**
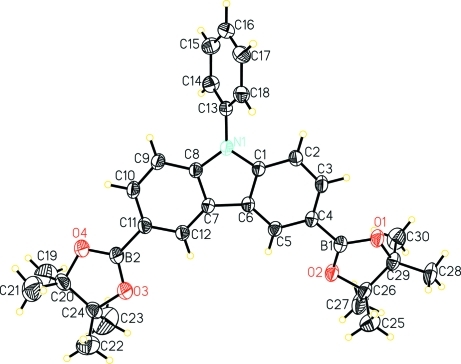
The molecular structure of the title molecule with the atom-numbering scheme. The displacement ellipsoids are drawn at the 30% probability level.

## supplementary crystallographic information

### Comment

Carbazole - based materials have been investigated for their electrical and optical properties. Especially, introduction of substituents on the 3, 6-positions of carbazole represents a possible approach for designing carbazole-based materials with electrogenerated chemiluminescence. The title compound is a key intermediates, which can be used to synthesize 9-phenylcarbazole derivatives with substituents at 3, 6-positions (Wong *et al.*, 2005, 2006; Rashidnadimi *et al.*, 2008).

The central structural element of the title compound is a carbazole moiety substituted with two pinacolbronic ester at 3, 6-positions and a phenyl attached to atom N9. The carbazole moiety is essentially planar (maximum deviation=0.057 Å). The carbazole plane is inclined to the phenyl ring planes at dihedral angle of 115.0 (3)°. The C—B distances fall in the range to 1.550 (4) Å, consistent with the literature (Allen *et al.*, 1987).The crystal packing is stabilized by van der Waals forces.

### Experimental

To a solution of 5,8-dibromo-1-phenylcarbazole (400 mg, 1 mmol) in THF (15 ml) at -78°C was added 1.87 ml (3 mmol) of *n*-butyllithium (1.6 *M* in hexane). The mixture was stirred at -78°C for 2 h. 0.4 ml (2 mmol) of 2-isopropoxy-4,4,5,5-tetramethyl-[1,3,2]-dioxaborolane was added rapidly to the solution, and the resulting mixture was warmed to room temperature and stirred for 8 h. The mixture was poured into water and extracted with dichloromethane. The organic extracts were washed with brine and dried over magnesium sulfate. The solvent was removed by rotary evaporation, and recrystallization was made in a mixture of *n*-pentane/hexane to afford 356 mg (72%) of product as a whitesolid. The structure was confirmed by FTIR, ^1^H NMR and MS. Single crystals suitable for crystallographic analysis were obtained by slow evaporation of a ethanol/dichloromethane (1:1v/v) solution.

### Refinement

All H atoms attached to C atoms were fixed geometrically and treated as riding with C—H = 0.96 Å(methyl) and C—H = 0.93 Å (aromatic) with *U*ĩso~(H) = 1.2*U*~eq~(aromatic) and *U*ĩso~(H) = 1.5*U*~eq~(methyl).

### Crystal data

**Table:**

C_30_H_35_B_2_NO_4_	*D*_x_ = 1.143 Mg m^−^^3^
*M**_r_* = 495.21	Melting point: 476 K
Orthorhombic, *P**b**c**a*	Mo *K*α radiation, λ = 0.71073 Å
Hall symbol: -P 2ac 2ab	Cell parameters from 10558 reflections
*a* = 13.974 (6) Å	θ = 2.1–27.5°
*b* = 11.935 (5) Å	µ = 0.07 mm^−^^1^
*c* = 34.494 (14) Å	*T* = 298 K
*V* = 5753 (4) Å^3^	Block, colourless
*Z* = 8	0.3 × 0.2 × 0.1 mm
*F*(000) = 2112	

### Data collection

**Table:**

Rigaku Mercury2 diffractometer	6553 independent reflections
Radiation source: fine-focus sealed tube	5170 reflections with *I* > 2σ(*I*)
graphite	*R*_int_ = 0.062
Detector resolution: 13.6612 pixels mm^-1^	θ_max_ = 27.5°, θ_min_ = 1.9°
ω scans	*h* = −18→18
Absorption correction: multi-scan (*CrystalClear*; Rigaku, 2005)	*k* = −15→15
*T*_min_ = 0.9, *T*_max_ = 1	*l* = −44→44
49645 measured reflections	

### Refinement

**Table:**

Refinement on *F*^2^	Secondary atom site location: difference Fourier map
Least-squares matrix: full	Hydrogen site location: inferred from neighbouring sites
*R*[*F*^2^ > 2σ(*F*^2^)] = 0.093	H-atom parameters constrained
*wR*(*F*^2^) = 0.242	*w* = 1/[σ^2^(*F*_o_^2^) + (0.0908*P*)^2^ + 3.0388*P*] where *P* = (*F*_o_^2^ + 2*F*_c_^2^)/3
*S* = 1.16	(Δ/σ)_max_ < 0.001
6553 reflections	Δρ_max_ = 0.72 e Å^−^^3^
342 parameters	Δρ_min_ = −0.52 e Å^−^^3^
15 restraints	Extinction correction: *SHELXL97* (Sheldrick, 2008), Fc^*^=kFc[1+0.001xFc^2^λ^3^/sin(2θ)]^-1/4^
Primary atom site location: structure-invariant direct methods	Extinction coefficient: 0.0056 (14)

### Special details

**Table:**

Geometry. All e.s.d.'s (except the e.s.d. in the dihedral angle between two l.s. planes) are estimated using the full covariance matrix. The cell e.s.d.'s are taken into account individually in the estimation of e.s.d.'s in distances, angles and torsion angles; correlations between e.s.d.'s in cell parameters are only used when they are defined by crystal symmetry. An approximate (isotropic) treatment of cell e.s.d.'s is used for estimating e.s.d.'s involving l.s. planes.
Refinement. Refinement of *F*^2^ against ALL reflections. The weighted *R*-factor *wR* and goodness of fit *S* are based on *F*^2^, conventional *R*-factors *R* are based on *F*, with *F* set to zero for negative *F*^2^. The threshold expression of *F*^2^ > σ(*F*^2^) is used only for calculating *R*-factors(gt) *etc*. and is not relevant to the choice of reflections for refinement. *R*-factors based on *F*^2^ are statistically about twice as large as those based on *F*, and *R*- factors based on ALL data will be even larger.

### Fractional atomic coordinates and isotropic or equivalent isotropic displacement parameters (Å^2^)

**Table:**

	*x*	*y*	*z*	*U*_iso_*/*U*_eq_	
O1	0.14754 (13)	0.80983 (19)	0.06118 (6)	0.0611 (6)	
O2	0.16305 (13)	0.7395 (2)	0.12237 (6)	0.0644 (6)	
C3	0.47750 (18)	0.7413 (2)	0.11098 (7)	0.0454 (6)	
N1	0.61593 (15)	0.8121 (2)	0.08578 (6)	0.0517 (6)	
C5	0.64049 (18)	0.7510 (2)	0.11878 (7)	0.0475 (6)	
C6	0.51688 (17)	0.8061 (2)	0.08084 (7)	0.0461 (6)	
C7	0.68001 (18)	0.8756 (2)	0.06208 (7)	0.0465 (6)	
C8	0.31920 (18)	0.7794 (2)	0.08579 (8)	0.0506 (6)	
C9	0.6547 (2)	0.6093 (3)	0.18283 (8)	0.0533 (7)	
C10	0.56499 (19)	0.6321 (2)	0.16660 (7)	0.0499 (6)	
H10A	0.5105	0.5994	0.1771	0.060*	
C11	0.36216 (19)	0.8401 (3)	0.05558 (8)	0.0562 (7)	
H11A	0.3230	0.8716	0.0367	0.067*	
C12	0.55668 (17)	0.7033 (2)	0.13485 (7)	0.0448 (6)	
C13	0.37813 (18)	0.7292 (2)	0.11331 (8)	0.0491 (6)	
H13A	0.3513	0.6874	0.1333	0.059*	
C14	0.7359 (2)	0.6605 (3)	0.16612 (8)	0.0607 (7)	
H14A	0.7956	0.6463	0.1769	0.073*	
C15	0.7300 (2)	0.9648 (3)	0.07770 (9)	0.0603 (7)	
H15A	0.7232	0.9829	0.1038	0.072*	
C16	0.73053 (19)	0.7308 (3)	0.13444 (8)	0.0587 (7)	
H16A	0.7851	0.7634	0.1239	0.070*	
C17	0.45997 (19)	0.8554 (2)	0.05254 (8)	0.0544 (7)	
H17A	0.4865	0.8968	0.0324	0.065*	
C18	0.6916 (2)	0.8491 (2)	0.02336 (8)	0.0557 (7)	
H18A	0.6590	0.7885	0.0127	0.067*	
C19	0.05223 (19)	0.8181 (3)	0.07865 (9)	0.0611 (8)	
O3	0.59377 (17)	0.4625 (2)	0.23038 (7)	0.0892 (7)	
C21	0.0605 (2)	0.7369 (3)	0.11389 (9)	0.0630 (8)	
C22	0.7514 (2)	0.9128 (3)	0.00057 (8)	0.0645 (8)	
H22A	0.7584	0.8952	−0.0255	0.077*	
B1	0.2088 (2)	0.7751 (3)	0.08979 (9)	0.0516 (7)	
O4	0.74200 (18)	0.5255 (2)	0.24089 (7)	0.0907 (7)	
C25	0.7291 (3)	0.4402 (3)	0.27042 (9)	0.0707 (9)	
B2	0.6640 (3)	0.5314 (4)	0.21859 (12)	0.0801 (8)	
C27	0.8004 (2)	1.0010 (3)	0.01569 (9)	0.0668 (8)	
H27A	0.8405	1.0436	0.0000	0.080*	
C28	−0.0211 (2)	0.7866 (4)	0.04848 (10)	0.0865 (12)	
H28A	−0.0135	0.7091	0.0417	0.130*	
H28B	−0.0123	0.8322	0.0258	0.130*	
H28C	−0.0842	0.7984	0.0587	0.130*	
C29	0.6219 (3)	0.4071 (3)	0.26585 (8)	0.0664 (8)	
C30	0.7904 (2)	1.0269 (3)	0.05424 (10)	0.0719 (9)	
H30A	0.8244	1.0867	0.0647	0.086*	
C31	0.0404 (3)	0.9398 (3)	0.09067 (14)	0.0974 (13)	
H31A	0.0437	0.9869	0.0681	0.146*	
H31B	0.0905	0.9599	0.1084	0.146*	
H31C	−0.0205	0.9495	0.1031	0.146*	
C32	0.0087 (3)	0.7720 (5)	0.15013 (10)	0.1018 (15)	
H32A	0.0196	0.7176	0.1702	0.153*	
H32B	−0.0587	0.7770	0.1449	0.153*	
H32C	0.0320	0.8437	0.1585	0.153*	
C33	0.7992 (4)	0.3483 (5)	0.26240 (19)	0.142 (2)	
H33A	0.8631	0.3775	0.2638	0.212*	
H33B	0.7880	0.3184	0.2370	0.212*	
H33C	0.7916	0.2899	0.2813	0.212*	
C34	0.5556 (4)	0.4521 (6)	0.29700 (15)	0.155 (3)	
H34A	0.5692	0.5298	0.3014	0.232*	
H34B	0.5651	0.4108	0.3206	0.232*	
H34C	0.4904	0.4440	0.2887	0.232*	
C35	0.0369 (3)	0.6160 (4)	0.10317 (14)	0.1071 (15)	
H35A	0.0581	0.5669	0.1235	0.161*	
H35B	0.0688	0.5967	0.0794	0.161*	
H35C	−0.0309	0.6082	0.0998	0.161*	
C36	0.6025 (4)	0.2839 (4)	0.26109 (18)	0.1287 (19)	
H36A	0.5353	0.2724	0.2569	0.193*	
H36B	0.6219	0.2448	0.2841	0.193*	
H36C	0.6377	0.2558	0.2393	0.193*	
C37	0.7538 (4)	0.4952 (5)	0.30874 (14)	0.135 (2)	
H37A	0.7156	0.5613	0.3121	0.202*	
H37B	0.8203	0.5152	0.3089	0.202*	
H37C	0.7411	0.4439	0.3295	0.202*	

### Atomic displacement parameters (Å^2^)

**Table:**

	*U*^11^	*U*^22^	*U*^33^	*U*^12^	*U*^13^	*U*^23^
O1	0.0418 (10)	0.0836 (14)	0.0579 (11)	0.0092 (9)	−0.0044 (8)	0.0047 (10)
O2	0.0427 (10)	0.0916 (16)	0.0588 (12)	0.0036 (10)	−0.0048 (8)	0.0047 (11)
C3	0.0420 (13)	0.0488 (14)	0.0454 (12)	0.0058 (11)	−0.0023 (10)	−0.0002 (10)
N1	0.0401 (11)	0.0684 (15)	0.0465 (11)	−0.0006 (10)	−0.0049 (9)	0.0088 (10)
C5	0.0423 (13)	0.0583 (15)	0.0418 (12)	0.0012 (11)	−0.0031 (10)	0.0024 (11)
C6	0.0404 (13)	0.0523 (14)	0.0455 (12)	0.0026 (11)	−0.0039 (10)	0.0000 (11)
C7	0.0415 (13)	0.0520 (14)	0.0462 (13)	0.0027 (11)	−0.0034 (10)	0.0052 (11)
C8	0.0408 (13)	0.0582 (16)	0.0528 (14)	0.0048 (11)	−0.0060 (11)	−0.0011 (12)
C9	0.0506 (15)	0.0639 (17)	0.0455 (13)	0.0036 (13)	−0.0062 (11)	0.0035 (12)
C10	0.0455 (14)	0.0585 (16)	0.0456 (13)	0.0022 (12)	−0.0009 (11)	0.0037 (11)
C11	0.0454 (14)	0.0678 (17)	0.0554 (15)	0.0095 (13)	−0.0103 (12)	0.0044 (13)
C12	0.0391 (12)	0.0517 (14)	0.0435 (12)	0.0031 (10)	−0.0028 (10)	−0.0026 (10)
C13	0.0406 (13)	0.0564 (15)	0.0502 (13)	0.0029 (11)	0.0003 (10)	0.0002 (12)
C14	0.0456 (15)	0.083 (2)	0.0539 (15)	0.0019 (14)	−0.0129 (12)	0.0097 (15)
C15	0.0638 (18)	0.0629 (18)	0.0543 (15)	−0.0040 (14)	−0.0014 (13)	−0.0069 (13)
C16	0.0425 (15)	0.082 (2)	0.0520 (14)	−0.0036 (13)	−0.0061 (11)	0.0098 (14)
C17	0.0473 (15)	0.0645 (17)	0.0514 (14)	0.0036 (12)	−0.0037 (11)	0.0093 (13)
C18	0.0602 (17)	0.0594 (16)	0.0476 (14)	−0.0033 (13)	−0.0051 (12)	−0.0003 (12)
C19	0.0394 (14)	0.080 (2)	0.0637 (17)	0.0072 (14)	−0.0045 (12)	−0.0096 (15)
O3	0.0719 (13)	0.1128 (17)	0.0830 (14)	−0.0171 (11)	−0.0228 (11)	0.0454 (13)
C21	0.0415 (14)	0.087 (2)	0.0602 (16)	−0.0044 (14)	−0.0053 (12)	−0.0049 (15)
C22	0.0708 (19)	0.075 (2)	0.0473 (14)	−0.0034 (16)	0.0023 (14)	0.0065 (14)
B1	0.0449 (16)	0.0575 (18)	0.0523 (16)	0.0068 (13)	−0.0057 (13)	−0.0075 (14)
O4	0.0729 (13)	0.1150 (17)	0.0841 (14)	−0.0185 (12)	−0.0307 (11)	0.0455 (13)
C25	0.085 (2)	0.0637 (19)	0.0635 (18)	−0.0032 (16)	−0.0241 (16)	0.0160 (15)
B2	0.0655 (14)	0.1032 (17)	0.0718 (15)	−0.0136 (13)	−0.0238 (12)	0.0382 (14)
C27	0.0590 (17)	0.073 (2)	0.0681 (19)	−0.0026 (16)	0.0018 (14)	0.0218 (16)
C28	0.0530 (19)	0.132 (3)	0.074 (2)	0.008 (2)	−0.0207 (16)	−0.007 (2)
C29	0.081 (2)	0.0681 (19)	0.0498 (15)	0.0003 (16)	−0.0061 (14)	0.0114 (14)
C30	0.072 (2)	0.0597 (18)	0.084 (2)	−0.0156 (16)	−0.0073 (17)	0.0009 (16)
C31	0.078 (2)	0.083 (3)	0.131 (4)	0.026 (2)	−0.009 (2)	−0.018 (2)
C32	0.0523 (19)	0.184 (5)	0.069 (2)	0.003 (2)	0.0066 (16)	−0.007 (3)
C33	0.094 (3)	0.110 (4)	0.221 (7)	0.029 (3)	−0.006 (4)	−0.002 (4)
C34	0.122 (4)	0.234 (8)	0.107 (4)	−0.007 (5)	0.032 (3)	−0.037 (4)
C35	0.102 (3)	0.087 (3)	0.132 (4)	−0.027 (2)	−0.024 (3)	0.007 (3)
C36	0.115 (4)	0.081 (3)	0.190 (6)	−0.020 (3)	−0.015 (4)	0.004 (3)
C37	0.161 (5)	0.153 (5)	0.090 (3)	−0.037 (4)	−0.045 (3)	−0.014 (3)

### Geometric parameters (Å, °)

**Table:**

O1—B1	1.371 (4)	C21—C32	1.504 (4)
O1—C19	1.465 (3)	C21—C35	1.526 (5)
O2—B1	1.361 (4)	C22—C27	1.361 (5)
O2—C21	1.463 (3)	C22—H22A	0.9300
C3—C13	1.398 (4)	O4—B2	1.336 (4)
C3—C6	1.408 (4)	O4—C25	1.452 (4)
C3—C12	1.452 (3)	C25—C33	1.497 (6)
N1—C5	1.395 (3)	C25—C37	1.516 (5)
N1—C6	1.396 (3)	C25—C29	1.556 (5)
N1—C7	1.429 (3)	C27—C30	1.373 (5)
C5—C16	1.390 (4)	C27—H27A	0.9300
C5—C12	1.415 (4)	C28—H28A	0.9600
C6—C17	1.389 (3)	C28—H28B	0.9600
C7—C18	1.382 (4)	C28—H28C	0.9600
C7—C15	1.383 (4)	C29—C36	1.505 (6)
C8—C13	1.392 (4)	C29—C34	1.517 (6)
C8—C11	1.404 (4)	C30—H30A	0.9300
C8—B1	1.550 (4)	C31—H31A	0.9600
C9—C10	1.399 (4)	C31—H31B	0.9600
C9—C14	1.412 (4)	C31—H31C	0.9600
C9—B2	1.550 (4)	C32—H32A	0.9600
C10—C12	1.391 (4)	C32—H32B	0.9600
C10—H10A	0.9300	C32—H32C	0.9600
C11—C17	1.383 (4)	C33—H33A	0.9600
C11—H11A	0.9300	C33—H33B	0.9600
C13—H13A	0.9300	C33—H33C	0.9600
C14—C16	1.379 (4)	C34—H34A	0.9600
C14—H14A	0.9300	C34—H34B	0.9600
C15—C30	1.384 (4)	C34—H34C	0.9600
C15—H15A	0.9300	C35—H35A	0.9600
C16—H16A	0.9300	C35—H35B	0.9600
C17—H17A	0.9300	C35—H35C	0.9600
C18—C22	1.376 (4)	C36—H36A	0.9600
C18—H18A	0.9300	C36—H36B	0.9600
C19—C28	1.508 (4)	C36—H36C	0.9600
C19—C31	1.519 (5)	C37—H37A	0.9600
C19—C21	1.559 (5)	C37—H37B	0.9600
O3—B2	1.343 (5)	C37—H37C	0.9600
O3—C29	1.445 (4)		
			
B1—O1—C19	107.0 (2)	O4—C25—C33	107.6 (4)
B1—O2—C21	107.6 (2)	O4—C25—C37	106.2 (3)
C13—C3—C6	119.2 (2)	C33—C25—C37	109.2 (4)
C13—C3—C12	133.8 (2)	O4—C25—C29	103.1 (2)
C6—C3—C12	107.0 (2)	C33—C25—C29	115.2 (3)
C5—N1—C6	108.4 (2)	C37—C25—C29	114.7 (4)
C5—N1—C7	126.1 (2)	O4—B2—O3	113.0 (3)
C6—N1—C7	125.3 (2)	O4—B2—C9	123.9 (3)
C16—C5—N1	129.1 (2)	O3—B2—C9	123.1 (3)
C16—C5—C12	121.8 (2)	C22—C27—C30	119.6 (3)
N1—C5—C12	109.0 (2)	C22—C27—H27A	120.2
C17—C6—N1	129.1 (2)	C30—C27—H27A	120.2
C17—C6—C3	121.8 (2)	C19—C28—H28A	109.5
N1—C6—C3	109.0 (2)	C19—C28—H28B	109.5
C18—C7—C15	119.6 (3)	H28A—C28—H28B	109.5
C18—C7—N1	120.3 (2)	C19—C28—H28C	109.5
C15—C7—N1	120.1 (2)	H28A—C28—H28C	109.5
C13—C8—C11	118.4 (2)	H28B—C28—H28C	109.5
C13—C8—B1	120.9 (3)	O3—C29—C36	107.8 (3)
C11—C8—B1	120.6 (2)	O3—C29—C34	105.7 (4)
C10—C9—C14	118.2 (2)	C36—C29—C34	108.2 (4)
C10—C9—B2	120.7 (3)	O3—C29—C25	103.4 (2)
C14—C9—B2	121.1 (3)	C36—C29—C25	115.6 (3)
C12—C10—C9	120.6 (3)	C34—C29—C25	115.3 (3)
C12—C10—H10A	119.7	C27—C30—C15	120.5 (3)
C9—C10—H10A	119.7	C27—C30—H30A	119.7
C17—C11—C8	123.1 (2)	C15—C30—H30A	119.7
C17—C11—H11A	118.4	C19—C31—H31A	109.5
C8—C11—H11A	118.4	C19—C31—H31B	109.5
C10—C12—C5	119.0 (2)	H31A—C31—H31B	109.5
C10—C12—C3	134.5 (2)	C19—C31—H31C	109.5
C5—C12—C3	106.5 (2)	H31A—C31—H31C	109.5
C8—C13—C3	120.3 (2)	H31B—C31—H31C	109.5
C8—C13—H13A	119.9	C21—C32—H32A	109.5
C3—C13—H13A	119.9	C21—C32—H32B	109.5
C16—C14—C9	122.9 (3)	H32A—C32—H32B	109.5
C16—C14—H14A	118.6	C21—C32—H32C	109.5
C9—C14—H14A	118.6	H32A—C32—H32C	109.5
C7—C15—C30	119.5 (3)	H32B—C32—H32C	109.5
C7—C15—H15A	120.2	C25—C33—H33A	109.5
C30—C15—H15A	120.2	C25—C33—H33B	109.5
C14—C16—C5	117.5 (3)	H33A—C33—H33B	109.5
C14—C16—H16A	121.2	C25—C33—H33C	109.5
C5—C16—H16A	121.2	H33A—C33—H33C	109.5
C11—C17—C6	117.2 (3)	H33B—C33—H33C	109.5
C11—C17—H17A	121.4	C29—C34—H34A	109.5
C6—C17—H17A	121.4	C29—C34—H34B	109.5
C22—C18—C7	119.8 (3)	H34A—C34—H34B	109.5
C22—C18—H18A	120.1	C29—C34—H34C	109.5
C7—C18—H18A	120.1	H34A—C34—H34C	109.5
O1—C19—C28	108.5 (3)	H34B—C34—H34C	109.5
O1—C19—C31	106.0 (3)	C21—C35—H35A	109.5
C28—C19—C31	110.7 (3)	C21—C35—H35B	109.5
O1—C19—C21	102.2 (2)	H35A—C35—H35B	109.5
C28—C19—C21	115.7 (3)	C21—C35—H35C	109.5
C31—C19—C21	112.9 (3)	H35A—C35—H35C	109.5
B2—O3—C29	109.7 (3)	H35B—C35—H35C	109.5
O2—C21—C32	107.4 (2)	C29—C36—H36A	109.5
O2—C21—C35	106.2 (3)	C29—C36—H36B	109.5
C32—C21—C35	111.1 (3)	H36A—C36—H36B	109.5
O2—C21—C19	102.5 (2)	C29—C36—H36C	109.5
C32—C21—C19	116.1 (3)	H36A—C36—H36C	109.5
C35—C21—C19	112.5 (3)	H36B—C36—H36C	109.5
C27—C22—C18	121.0 (3)	C25—C37—H37A	109.5
C27—C22—H22A	119.5	C25—C37—H37B	109.5
C18—C22—H22A	119.5	H37A—C37—H37B	109.5
O2—B1—O1	113.3 (3)	C25—C37—H37C	109.5
O2—B1—C8	123.5 (2)	H37A—C37—H37C	109.5
O1—B1—C8	123.2 (3)	H37B—C37—H37C	109.5
B2—O4—C25	109.8 (3)		
			
C6—N1—C5—C16	178.1 (3)	B1—O1—C19—C21	−23.4 (3)
C7—N1—C5—C16	−5.1 (5)	B1—O2—C21—C32	−143.9 (3)
C6—N1—C5—C12	1.1 (3)	B1—O2—C21—C35	97.0 (3)
C7—N1—C5—C12	177.9 (2)	B1—O2—C21—C19	−21.2 (3)
C5—N1—C6—C17	178.7 (3)	O1—C19—C21—O2	26.7 (3)
C7—N1—C6—C17	1.9 (4)	C28—C19—C21—O2	144.4 (3)
C5—N1—C6—C3	0.3 (3)	C31—C19—C21—O2	−86.7 (3)
C7—N1—C6—C3	−176.5 (2)	O1—C19—C21—C32	143.5 (3)
C13—C3—C6—C17	−2.2 (4)	C28—C19—C21—C32	−98.9 (4)
C12—C3—C6—C17	179.9 (2)	C31—C19—C21—C32	30.1 (4)
C13—C3—C6—N1	176.3 (2)	O1—C19—C21—C35	−87.0 (3)
C12—C3—C6—N1	−1.6 (3)	C28—C19—C21—C35	30.7 (4)
C5—N1—C7—C18	119.4 (3)	C31—C19—C21—C35	159.6 (3)
C6—N1—C7—C18	−64.3 (4)	C7—C18—C22—C27	0.7 (5)
C5—N1—C7—C15	−61.3 (4)	C21—O2—B1—O1	7.2 (3)
C6—N1—C7—C15	115.0 (3)	C21—O2—B1—C8	−174.5 (3)
C14—C9—C10—C12	0.1 (4)	C19—O1—B1—O2	11.4 (3)
B2—C9—C10—C12	179.1 (3)	C19—O1—B1—C8	−166.9 (3)
C13—C8—C11—C17	−2.1 (4)	C13—C8—B1—O2	10.8 (4)
B1—C8—C11—C17	174.0 (3)	C11—C8—B1—O2	−165.2 (3)
C9—C10—C12—C5	1.0 (4)	C13—C8—B1—O1	−171.1 (3)
C9—C10—C12—C3	177.9 (3)	C11—C8—B1—O1	12.9 (4)
C16—C5—C12—C10	−1.6 (4)	B2—O4—C25—C33	113.9 (4)
N1—C5—C12—C10	175.6 (2)	B2—O4—C25—C37	−129.2 (4)
C16—C5—C12—C3	−179.3 (3)	B2—O4—C25—C29	−8.3 (4)
N1—C5—C12—C3	−2.1 (3)	C25—O4—B2—O3	3.3 (5)
C13—C3—C12—C10	7.6 (5)	C25—O4—B2—C9	−176.8 (4)
C6—C3—C12—C10	−174.9 (3)	C29—O3—B2—O4	3.8 (5)
C13—C3—C12—C5	−175.2 (3)	C29—O3—B2—C9	−176.1 (4)
C6—C3—C12—C5	2.2 (3)	C10—C9—B2—O4	−164.1 (4)
C11—C8—C13—C3	1.1 (4)	C14—C9—B2—O4	14.9 (6)
B1—C8—C13—C3	−174.9 (2)	C10—C9—B2—O3	15.8 (6)
C6—C3—C13—C8	0.9 (4)	C14—C9—B2—O3	−165.3 (4)
C12—C3—C13—C8	178.1 (3)	C18—C22—C27—C30	0.2 (5)
C10—C9—C14—C16	−0.7 (5)	B2—O3—C29—C36	−131.5 (4)
B2—C9—C14—C16	−179.7 (3)	B2—O3—C29—C34	113.0 (4)
C18—C7—C15—C30	0.6 (4)	B2—O3—C29—C25	−8.6 (4)
N1—C7—C15—C30	−178.7 (3)	O4—C25—C29—O3	9.9 (3)
C9—C14—C16—C5	0.1 (5)	C33—C25—C29—O3	−107.1 (4)
N1—C5—C16—C14	−175.6 (3)	C37—C25—C29—O3	124.8 (4)
C12—C5—C16—C14	1.0 (4)	O4—C25—C29—C36	127.5 (4)
C8—C11—C17—C6	0.8 (4)	C33—C25—C29—C36	10.5 (5)
N1—C6—C17—C11	−176.8 (3)	C37—C25—C29—C36	−117.6 (4)
C3—C6—C17—C11	1.4 (4)	O4—C25—C29—C34	−105.0 (4)
C15—C7—C18—C22	−1.1 (4)	C33—C25—C29—C34	138.0 (5)
N1—C7—C18—C22	178.1 (3)	C37—C25—C29—C34	9.9 (5)
B1—O1—C19—C28	−146.1 (3)	C22—C27—C30—C15	−0.7 (5)
B1—O1—C19—C31	95.0 (3)	C7—C15—C30—C27	0.4 (5)
